# Supplementary material for: Seizure Detection: A Low Computational Effective Approach without Classification Methods
Source: Sensors (Basel). 2022 Nov 3;22(21):8444. doi: 10.3390/s22218444 (PMC9657642; doi:10.3390/s22218444)
Supplement: Supplementary file 1 [file sensors-22-08444-s001.zip › sensors-1740238-supplementary.pdf]

Steps to correctly execute our code

1) Unzip the content of the zipped file into a folder accessible to MATLAB
2) Open the script "process_all_chs.m"
3) Select the file to process at line 13, see comments lines 9-11 for included files
    a. The software runs correctly for the entire dataset
       https://physionet.org/content/chbmit/1.0.0/
       However, other files not included in the examples need to be manually downloaded
4) Execute the script "process_all_chs.m"
5) If the algorithm does not detect a seizure it displays "No seizures detected... and the result figure will have a flat line on zero. If a seizure is detected its duration will be marked by the value of one on the plot, the algorithm will display "Seizures detected..."
